# Adverse events of ursodeoxycholic acid: a real-world pharmacovigilance study using FAERS (2004–2023)

**DOI:** 10.3389/fmed.2025.1574308

**Published:** 2025-09-02

**Authors:** Yahui Ni, Xin Guan, Xiaoxue Gao, Ya Wang, Dongyuan Qin, Zhangtao Shan, Na Wang

**Affiliations:** ^1^Department of Gastroenterology, Shanxi Bethune Hospital, Shanxi Academy of Medical Sciences, Third Hospital of Shanxi Medical University, Tongji Shanxi Hospital, Taiyuan, China; ^2^Department of Cardiology, Shanxi Bethune Hospital, Shanxi Academy of Medical Sciences, Tongji Shanxi Hospital, Third Hospital of Shanxi Medical University, Taiyuan, China

**Keywords:** ursodeoxycholic acid, adverse events, FAERS database, pharmacovigilance study, disproportionality analysis

## Abstract

**Background:**

Ursodeoxycholic acid (UDCA) has been widely used in the treatment of hepatobiliary disorders and its clinical application is more and more extensive. However, to our knowledge, there are currently no clinical and scientific studies on the safety of UDCA based on large populations. In this study, UDCA-related adverse events (AEs) were evaluated through data mining based on the FDA Adverse Event Reporting System (FAERS) database.

**Methods:**

The AE reports induced by UDCA as the primary suspected drug were extracted from the FAERS database. Disproportionality analysis was performed to explore potential AE signals of UDCA using four robust algorithms, including reporting odds ratio (ROR), the proportional reporting ratio (PRR), the Bayesian confidence propagation neural network (BCPNN), and the Empirical Bayesian geometric mean (EBGM). The difference in UDCA-associated AE signals was also investigated concerning sex.

**Results:**

A total of 1,651 AEs were identified to be associated with UDCA. Common AEs consistent with the drug insert included diarrhea or loose stools, right upper abdominal pain, rash, and so on. Several unexpected AEs, such as interstitial lung disease and pancytopenia, were also identified. UDCA-related AEs affected 27 system organ classes (SOCs), and the signal intensity showed gender differences.

**Conclusion:**

This study investigated AEs associated with UDCA in both SOC and preferred terms (PTs) levels, providing valuable insights to the comprehensive landscape of AEs caused by UDCA. The results of this study help optimize the clinical use of UDCA and reduce its potential side effects, promoting its safe use in clinical application.

## 1 Introduction

Ursodeoxycholic acid (UDCA), is one of the normal components of human bile, accounting for 1%–3% of the bile tank content (1, 2). Under physiological conditions, UDCA can reduce the saturation of cholesterol in bile and promote cholesterol dissolution (3, 4). Under pathological conditions, UDCA can promote bile acid metabolism and play an important role in immune regulation, so it is mainly used in the treatment of cholestatic liver disease, cholesterol gallstones and bile reflux gastritis (5). In recent years, many studies have found that UDCA also has a good therapeutic effect on a variety of extrahepatic diseases, such as coronary heart disease, diabetes, heart failure, etcextrahepatic diseases and so on. So the role and safety of UDCA are increasingly valued by clinical workers (6).

Many phase III, phase IV clinical studies and randomized controlled trials have shown that UDCA has high safety and few AEs in long-term treatment, therefore it has a good patient adherence (7). However, due to the limitations of clinical studies and randomized controlled trials, such as strict sample selection criteria, relatively insufficient sample size and limited follow-up time, a comprehensive analysis is needed to explore the relationship between UDCA and its possible adverse events. The FDA Adverse Event Reporting System (FAERS) was established in the United States in 2012 and records a large number of AEs and medication errors related to the use of drugs which have been approved by FDA and therapeutic biologics all over the world. It has been widely used for the investigation of drug safety information (8, 9), so in this study, we aims to identify known and novel adverse events related to UDCA using the FAERS database, and with an additional focus on sex-specific signal differences, in order to provide recommendations for the safe clinical usage of UDCA.

## 2 Materials and methods

### 2.1 Data source and preprocessing

The data in FAERS database was primarily sourced from Legacy AERS, collected and preprocessed by SAS and MySQL. It contains reports of AEs submitted by healthcare professionals, consumers, manufacturers, and so on. The FAERS datasets consists seven data files: DEMO (demographics and administrative information), DRUG (drug information), REAC (adverse event coding), OUCT (patient outcomes), RPSR (report sources), THER (therapy start and end dates for reported drugs), and INDI (indications for use) (10). The FAERS database is open access and updated quarterly. Up to now, it has been used by many researchers to explore unexpected AEs that have not been mentioned and described in drug inserts, and has become a valuable resource for early detection and monitoring of drug safety issues (11).

**FIGURE 1:**
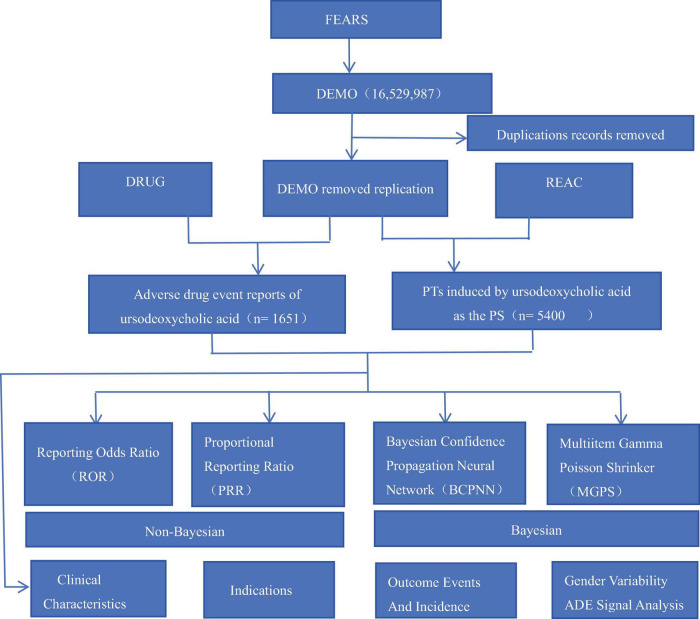
The flowchart of the data analysis.

In this study, we conducted a retrospective, disproportionality, and pharmacovigilance study to investigate AEs associated with UDCA on the basis of FAERS. The search time was set between the second quarter of 2004 and the fourth quarter of 2023. The duplicated reports were removed according to FDA recommended criteria to improve the reliability of the results. Finally, a total of 1,651 DEMO cases, 7,104 DRUG cases, and 5,400 REAC records were obtained (Figure 1).

### 2.2 Drug exposure

Based on the impact on AEs, the drugs in FAERS database were classified into four categories: PS (primary suspect), SS (second suspect), C (concomitant), and I (interacting). To improve accuracy, in this study, only UDCA as PS drugs were retained. The Medical Dictionary for Regularly Activities (MedDRA) was employed to classify adverse reactions. It contains multiple structural hierarchies, system organ class (SOC) was its highest level of terminology (12). According to MedDRA, the screened adverse reactions were subsequently mapped into system organ classes (SOCs) and preferred terms (PTs).

### 2.3 Pharmacovigilance study

The statistical analysis employed descriptive statistical methods to ADRs associated with UDCA. To find pairs of ADRs associated with UDCA, we employed several algorithms for metrics. Disproportionation analysis was a tool for hypothesizing possible causal relationships between drugs and adverse reactions, with subsequent clinical assessment of underlying case reports (13). It including two non-Bayesian methods: reporting odds ratio (ROR) and the proportional reporting ratio (PRR); and two Bayesian methods: the Bayesian confidence propagation neural network (BCPNN), and the Empirical Bayesian geometric mean (EBGM) (14). The advantage of the non-Bayesian method is simple to calculate and has high sensitivity, but when the number of adverse events is small, the likelihood of false positives is high (15). The Bayesian approach is stable. It accounts for the uncertainty in the disproportionate rate when the reports are small, reduces the likelihood of false positives, and is used for pattern recognition in higher dimensions, but it is computationally complex and has a relatively lagged signal detection time (16). Therefore, in this study, we used all the four methods to investigate the potential signals between UDCA and AEs. In addition, a gender disambiguation analysis was also performed to further explore gender differences in drug-related AEs. The meaning of a, b, c, and d were shown in Table 1. Signals that satisfy all four algorithms at the same time were considered statistically significant. The specific formulas were as follows:

**TABLE 1:** Four grid table.

Drug	Target AEs	Non-target AEs	Total
Ursodeoxycholic acid	a	b	a+b
Non-ursodeoxycholic acid	c	d	c+d
Total	a+c	b+d	N = a+b+c+d

Equation: a, total number of reports containing both the target drug and target adverse drug reaction; b, total number of reports containing other adverse drug reaction of the target drug; c, total number of people with target AEs after exposure to non-ursodeoxycholic acid; d, total number of people who developed non-targeted AEs after being exposed to non-ursodeoxycholic acid.

#### 2.3.1 ROR algorithm


$$\mathrm{ROR}=\frac{\mathrm{ad}}{\mathrm{bc}}$$

$$95\%\,\mathrm{CI}=e^{\ln\,\left(\mathrm{ROR}\right)\pm 1.96\,\sqrt{\left(\frac{1}{a}+\frac{1}{b}+\frac{1}{c}+\frac{1}{d}\right)}}$$

If the lower limit of 95% CI > 1 and a ≥ 3, the ROR is a striking signal.

#### 2.3.2 PRP algorithm


$$\mathrm{PRR}=\frac{a/\left(a+b\right)}{c/\left(c+d\right)}$$

$$\chi^{2}=\frac{{\left(\mathrm{ad}-\mathrm{bc}\right)}^{2}\,\left(a+b+c+d\right)}{\left(a+b\right)\,\left(a+c\right)\,\left(c+d\right)\,\left(b+d\right)}$$

If PRR ≥2, χ^2^ ≥ 4, a ≥3, *p* < 0.05, the PRR is a striking signal.

#### 2.3.3 BPCNN algorithm


$$\mathrm{IC}=\log_{2}\,\frac{a\,\left(a+b+c+d\right)}{\left(a+b\right)\,\left(a+c\right)}\,\mathrm{IC}-2\,S\,D=E\,\left(\mathrm{IC}\right)-2\,\sqrt{V\,\left(\mathrm{IC}\right)}$$

If IC-2SD > 0 (IC-2SD: the lower bound of 95% CI), the BPCNN is a positive signal. The signal intensity was strikingly correlated with the IC-2SD value.

#### 2.3.4 EBGM algorithm


$$\mathrm{EBGM}=\frac{a\,\left(a+b+c+d\right)}{\left(a+c\right)\,\left(a+b\right)}$$

$$95\%\,\mathrm{CI}=e^{\ln\,\left(\mathrm{EBGM}\right)\pm 1.96\,\sqrt{\left(\frac{1}{a}+\frac{1}{b}+\frac{1}{c}+\frac{1}{d}\right)}}$$

If EBGM05 > 2 (EBGM05: the lower bound of 95% CI), the BPCNN is a striking signal.

### 2.4 Statistical analysis

A descriptive analysis of AEs associated with UDCA was performed. Kruskal-Wallis test and Dunn’s test were used to assess the difference in values between the different groups. SAS, MySQL, WPS EXCEL and R software tools were employed to perform data processing and analysis.

## 3 Results

### 3.1 Descriptive analysis

FDA Adverse Event Reporting System database were reported spontaneously and submitted from multiple sources, so the data is inevitably prone to omissions, missing, repeated reporting, incomplete reporting, non-standard reporting, and so on, which may lead to bias in the study results (17). In this study, a total of 16,529,987 reports were extracted from FAERS database, covering the period between the second quarter of 2004 and fourth quarter of 2023. After remove duplications, a total of 1,651 AEs associated with UDCA were finally identified. The characteristics of AEs associated with UDCA were shown in Table 2. In terms of gender, females (*n* = 748, 45.3%) accounted for a higher proportion compared to males (*n* = 425, 25.7%). It might be attributed to the fact that the major indications for UDCA such as primary biliary cholangitis, intrahepatic cholestasis during pregnancy were more common in females. In terms of age composition, patients aged 18–65 years were more likely to experience AEs than those in other age groups, accounting for 28.5% (*n* = 470). In terms of weight, patients weighted between 50 and 100 kg constituted a major portion (*n* = 108, 6.5%). The majority of reports were provided by consumers, with health professionals accounting for around a quarter of the submissions (*n* = 459, 27.8%). In terms of geography, the country that submitted the most reports was Japan (*n* = 496, 30%), followed by the United States (*n* = 457, 27.7%), Netherlands (*n* = 121, 7.4%), France (*n* = 120, 7.2%), and Canada (*n* = 92, 5.6%). China reported only 31 cases, accounting for 1.9%, suggesting that Chinese clinical workers should pay more attention to evaluate and identify the potential AEs of UDCA during its application. The majority of serious outcomes was hospitalization (*n* = 418, 22%), followed by death (*n* = 249, 13.1%), life-threatening events (*n* = 54, 2.8%), and disability (*n* = 20, 1.1%).

**TABLE 2:** The characteristics of case reports associated with ursodeoxycholic acid as primary suspected drug in FDA Adverse Event Reporting System (FAERS) (from 2004 Q2 to 2023 Q4).

Characteristics	Case number (*n*)	Case percentage (%)
**Total number of reports *N* = 1,651**		
**Gender**		
Male	425	25.7
Female	748	45.3
Unknown	478	29.0
**Weight (kg)**		
< 50 kg	72	4.4
50–100 kg	108	6.5
> 100 kg	10	0.6
Unknown	1,461	88.5
**Age (year)**		
< 18	72	4.4
18–65	470	28.5
65–85	229	13.9
> 85	13	0.8
Unknown	868	52.6
**Reported country (top five)**		
Japan	496	30.0
United States	457	27.7
Netherlands	121	7.4
France	120	7.2
Canada	92	5.6
**Outcomes**		
Hospitalization (HO)	418	22
Death (DE)	249	13.1
Life-threatening (LT)	54	2.8
Disability (DS)	20	1.1
**Reported person**		
Consumer	860	52.1
Healthy professional	459	27.8
Unknown	332	20.1

**FIGURE 2:**
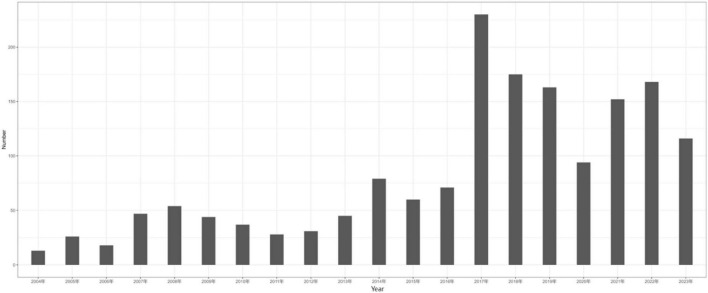
Year chart of adverse reactions reported by ursodeoxycholic acid.

Primary biliary cholangitis was the most common indication for UDCA, followed by cholestasis, choledocholithiasis, intrahepatic cholestasis of pregnancy and sclerosing cholangitis. The annual distribution of AEs associated with UDCA was shown in Figure 2. The results indicated that the number of UDCA-related AE reports was lowest in 2004 with 13 reports, and increased sharply 2017 and remained the highest in 2017–2019, the number was highest in 2017 with 240 reports. These results highlighted that UDCA had a more and more important role in clinical application. AEs associated with UDCA need to be closely monitored in the future.

### 3.2 Signal detection at the SOC level

Ursodeoxycholic acid-related AEs occurred in 27 SOCs in the FAERS database, indicating that the UDCA-related AEs were common in multiple organs (Supplementary Table 1). The top 10 SOCs with the highest frequency were general disorders and administration site conditions (*n* = 733); hepatobiliary disorders (*n* = 598); gastrointestinal disorders (*n* = 568); injury, poisoning and procedural complications (*n* = 541); investigations (*n* = 510); skin and subcutaneous tissue disorders (*n* = 362); pregnancy, puerperium and perinatal conditions (*n* = 264); respiratory, thoracic and mediastinal disorders (*n* = 215); nervous system disorders (*n* = 211); infections and infestations (*n* = 195). Among these, hepatobiliary disorders, gastrointesinal disorders, skin and subcutaneous tissue disorders were frequently encountered in our hospital applications and have been mentioned in the drug insert, further confirming the reliability of our findings. However, respiratory system disorders, nervous system disorders, infections and infestations, and blood and lymphatic system disorders were other new and valuable AEs at the SOC levels, which have not been mentioned in the drug insert of UDCA. We further used all the four disproportionality analysis algorithms, including ROR, PRR, BCPNN, and EBGM, to detect the signal values. SOCs that meet the detection criteria of at least one algorithm include: hepatobiliary disorders (*n* = 598); gastrointestinal disorders (*n* = 568); poisoning and procedural complications (*n* = 541); investigations (*n* = 510); skin and subcutaneous tissue disorders (*n* = 362); pregnancy, puerperium and perinatal conditions (*n* = 264); blood and lymphatic system disorders (*n* = 152); renal and urinary disorders (*n* = 116); surgical and medical procedures (*n* = 105); immune system disorders (*n* = 68); congenital, familial and genetic disorders (*n* = 54); endocrine disorders (*n* = 23) (Supplementary Table 2). There were three SOCs that simultaneously satisfied all the four algorithms: hepatobiliary disorders (*n* = 598); pregnancy, puerperium and perinatal conditions (*n* = 264); congenital, familial and genetic disorders (*n* = 54) (Table 3).

**TABLE 3:** The signal strength of adverse events (AEs) associated with ursodeoxycholic acid at the system organ class (SOC) level in the FDA Adverse Event Reporting System (FAERS) database (satisfy all the four algorithms).

SOC name	Case numbers	ROR (95% CI)	PRR (95% CI)	Chi square	IC (IC025)	EBGM (EBGM05)
Hepatobiliary disorders	598	13.51 (12.41–14.71)	12.13	6154.41	1.93	11.13
Pregnancy, puerperium and perinatal conditions	264	11.66 (10.30–13.20)	11.14	2444.61	1.81	9.83
Congenital, familial and genetic disorders	54	3.18 (2.43–4.16)	3.16	79.80	0.01	2.41

### 3.3 Signal detection at the PT level

In this study, all the four disproportionality analysis algorithms were employed to compute the signal values of UDCA-related AEs in the FAERS database to screen for meaningful AEs. The results showed that 187, 182, 881, and 395 UDCA-related AEs could be obtained by using ROR, PRR, BCPNN and EBGM, respectively (Supplementary Tables 3–6). Among them, when using EBGM, which was the most stringent algorithm, the top 5 AEs were pruritus (*n* = 105), maternal exposure during pregnancy (*n* = 79), interstitial lung disease (*n* = 58), liver disorder (*n* = 57), and alanine aminotransferase increased (*n* = 43), according to the number of reports. The top 30 UDCA-related AEs that met all of the four algorithms simultaneously were shown in Table 4 (ranked by the number of reports).

**TABLE 4:** The signal values of the top 30 adverse events (AEs) associated with ursodeoxycholic acid (UDCA) at the preferred terms (PTs) level simultaneously satisfy all the four algorithms, and ranked by case numbers.

PTs	SOC name	Case numbers	EBGM (EBGM05)
Pruritus	Skin and subcutaneous tissue disorders	105	3.27 (2.78)
Interstitial lung disease	Respiratory, thoracic and mediastinal disorders	58	14.20 (11.44)
Liver disorder	Hepatobiliary disorders	57	14.93 (12.00)
Fetal exposure during pregnancy	Injury, poisoning and procedural complications	40	5.77 (4.45)
Premature labor	Pregnancy, puerperium and perinatal conditions	40	14.53 (11.20)
Hepatic failure	Hepatobiliary disorders	32	11.65 (8.71)
Jaundice	Hepatobiliary disorders	29	11.51 (8.48)
Hepatic function abnormal	Hepatobiliary disorders	27	8.61 (6.28)
Pancytopenia	Blood and lymphatic system disorders	23	4.79 (3.40)
Venoocclusive liver disease	Hepatobiliary disorders	23	55.16 (39.11)
Anemia	Blood and lymphatic system disorders	21	3.22 (2.85)
Hepatic encephalopathy	Nervous system disorders	18	21.11 (14.32)
Multiple organ dysfunction syndrome	General disorders and administration site conditions	18	4.53 (3.08)
Drug eruption	Skin and subcutaneous tissue disorders	17	11.43 (7.67)
Respiratory tract malformation	Congenital, familial and genetic disorders	14	407.88 (260.41)
Pre-eclampsia	Pregnancy, puerperium and perinatal conditions	12	28.79 (17.91)
Lung cyst	Respiratory, thoracic and mediastinal disorders	11	306.22 (185.13)
Erythema multiforme	Skin and subcutaneous tissue disorders	10	11.97 (7.12)
Tubulointerstitial nephritis	Renal and urinary disorders	9	5.09 (2.95)
Agranulocytosis	Blood and lymphatic system disorders	9	5.90 (3.41)
Coagulopathy	Blood and lymphatic system disorders	8	5.21 (2.91)
Hypoproteinaemia	Metabolism and nutrition disorders	8	11.05 (6.18)
Cytomegalovirus infection	Infections and infestations	7	4.86 (2.62)
Iron deficiency anemia	Blood and lymphatic system disorders	7	8.64 (4.65)
Eosinophil count increased	Blood and lymphatic system disorders	7	9.75 (5.24)
Iactation disorder	Reproductive system and breast disorders	7	153.08 (81.90)
Pemphigoid	Skin and subcutaneous tissue disorders	6	10.13 (5.18)
Toxic skin eruption	Skin and subcutaneous tissue disorders	6	6.70 (3.43)
Toxic epidermal necrolysis	Skin and subcutaneous tissue disorders	6	4.58 (2.34)
Vanishing bile duct syndrome	Hepatobiliary disorders	6	74.16 (37.85)

Interestingly, in addition to diarrhea, pruritus and other AEs that have been mentioned in the drug insert, we also found some AEs that have not been mentioned in the instructions. These AEs may have respiratory effects, such as interstitial lung disease (*n* = 58); may have effects on the function of the reproductive system, such as lactation disorder (*n* = 7); and may affect the blood system, such as causing anemia (*n* = 21), coagulopathy (*n* = 8), pancytopenia (*n* = 23). More interestingly, several unexpected AEs, such as ileus (*n* = 5), pancreatitis acute (*n* = 6), cytomegalovirus infection (*n* = 7), and so on, were also identified. These AEs were also needed to be further monitored.

### 3.4 Sex differences for UDCA-related AEs

For the evaluation of drug safety, it is also important for us to consider the possibility of gender differences, which can help to manage AEs more precisely. Therefore, in this study, all the four disproportionality analysis algorithms were used to detect signal values to minimize the potential confusing effect of baseline information. The results showed that a total of 55 UDCA-related AEs were associated with males (Supplementary Table 7) and 74 UDCA-related AEs were associated with females (Supplementary Table 8).

Comparing the AEs of different genders, we found that many AEs could occur in both men and women, such as interstitial lung disease [*n* = 58, EBGM: 14.20 (11.44)], drug eruption [*n* = 17, EBGM: 11.43 (7.67)], erythema multiforme [*n* = 10, EBGM: 11.97 (7.12)], toxic epidermal necrolysis [*n* = 6, EBGM: 4.58 (2.34)], hepatic function abnormal [*n* = 27, EBGM: 8.61 (6.28)], hepatic failure [*n* = 32, EBGM: 11.65 (8.71)], tubulointerstitial nephritis [*n* = 9, EBGM: 5.09 (2.95)], pemphigoid [*n* = 6, EBGM: 10.13 (5.18)], coagulopathy [*n* = 8, EBGM: 5.21 (2.91)], and so on. However, there were some AEs that were only seen in one sex. For example, there were some AEs that could only be seen in males such as pancytopenia [*n* = 15, EBGM: 8.58 (5.61)], venoocclusive liver disease [*n* = 9, EBGM: 59.88 (34.55)], multiple organ dysfunction syndrome [*n* = 9, EBGM: 5.72 (3.31)], angioedema [n = 8, EBGM: 5.39 (3.01)], cytomegalovirus infection [*n* = 5, EBGM:9.41 (4.51)], human herpesvirus 6 infection [*n* = 4, EBGM:74.44 (32.67)], hepatic steatosis [*n* = 4, EBGM: 7.97 (3.50)], ileus [*n* = 3, EBGM: 7.74 (3.00)], pneumocystis jirovecii pneumonia [*n* = 3, EBGM: 7.65 (2.96)], bile duct cancer [*n* = 3, EBGM: 65.53 (25.33)], hypercalcaemia [*n* = 3, EBGM: 9.65 (3.74)], and so on. Meanwhile, pruritus [*n* = 75, EBGM: 4.31 (3.56)], thrombocytopenia [*n* = 13, EBGM: 3.40 (2.16)], agranulocytosis [*n* = 7, EBGM: 11.08 (5.95)], calculus urinary [*n* = 5, EBGM: 103.84 (49.66)], haematuria [*n* = 5, EBGM: 5.37 (2.57)], iron deficiency anemia [*n* = 4, EBGM: 9.42 (4.15)], type I hypersensitivity [*n* = 4, EBGM: 32.33 (14.21)], hypoproteinaemia [*n* = 4, EBGM: 15.86 (6.97)], vasculitis[*n* = 4, EBGM: 8.23 (3.62)], cerebral infarction [*n* = 4, EBGM: 5.01 (2.21)], schizophrenia [*n* = 4, EBGM: 11.23 (4.94)], pneumonia bacterial [*n* = 3, EBGM: 11.95 (4.63)] etc., could only be seen in females.

## 4 Discussion

Ursodeoxycholic acid is a naturally occurring bile acid that is present in small amounts in humans. As is known to all, it is the first-line therapy for cholestatic liver disease, such as primary biliary cholangitis, intrahepatic cholestasis of pregnancy and choledocholithiasis (18). Autoimmune hepatitis (AIH)-PBC, AIH-primary sclerosing cholangitis (PSC), AIH-PBC-PSC overlap syndrome are also beneficial (19–21). In addition, it has been confirmed that UDCA can be used for various extrahepatic diseases, such as heart failure, stroke, pneumonia, Corona Virus Disease 2019 (COVID-19), neurodegenerative Disease, diabetes and so on (22), the role of UDCA in clinical practice has been paid more and more attention. Possible mechanisms involve increasing the nuclear factor erythroid2 related factor 2 (Nrf2) levels, suppressing miR21/PTEN/AKT/mTOR signaling pathway and NF-κB signaling pathway, inhibiting the production of reactive oxygen species and pro-inflammatory cytokines. Ultimately, it exerts antioxidant, anti-inflammatory, anti-apoptosis and cell protective role (23–25).

So understanding the drug safety of ursodeoxycholic acid is becoming more and more important, known side effects of UDCA are few and most of them are relatively mild (26). However, larger studies in the real clinical world have not been reported, and information obtained from clinical trials may not precisely depict the actual circumstances in the real world. It is essential to gather pharmacovigilance data from post-marketing systems that report adverse events, which would greatly enhance drug specifications. Therefore, in this study, we accessed the publicly available database (the FAERS database) to identify UDCA-related AEs that had not been recorded in the drug instructions, and further conducted gender difference analysis, with the aim of providing more comprehensive and objective recommendations for the safe use of UDCA. The main results have been summarized as follows:

### 4.1 Gastrointestinal system

Loose stool or diarrhea was the most common gastrointestinal system AEs in UDCA treatment, with an incidence about 2%–9%, this is consistent with our research findings. It was dose-dependent and could be stopped after reducing or stopping UDCA treatment. The mechanism may be that gut bacteria convert UDCA into goosedeoxycholic acid, which acts as a secretagogue in the colon and causes diarrhea (27, 28). Abdominal pain upper, nausea, vomiting, abdominal distension and constipation were other digestive AEs shown in our study, which have been reported in the previous articles, possible mechanism may be UDCA can directly stimulate gastrointestinal mucosa and accelerate gastrointestinal peristalsis (29, 30). In addition, our study also reported some other gastrointestinal system AEs that have not been mentioned in the drug insert or previous studies, such as stomatitis, aphthal ulcer, cheilitis, tongue discolouration, plicated tongue, hypoaesthesia oral, and so on. Further exploration of the mechanism revealed that the imbalance of T lymphocyte subsets can lead to oral mucosal lesions. For example, patients with aphthal ulcer usually had an increased level of total T lymphocytes and CD8^+^T lymphocytes, a reduced proportion of CD4^+^T lymphocytes and CD4^+^/CD8^+^T lymphocytes (31). Previous studies have shown that UDCA could regulate the proportion of T lymphocytes in patients, causing a decrease in the count of CD4^+^T lymphocytes, CD3^+^CD4^+^T lymphocytes and a decrease in the proportion of CD4^+^/CD8^+^T lymphocytes, promoting the secretion of Th2 cytokines, which may be related to secondary oral mucosal lesions (32). Therefore, patients treated with UDCA for a long time should be wary of oral mucosal damage and oral microenvironment changes. Acute pancreatitis and ileus are other AEs that reported firstly by us, but the mechanism of their occurrence is still unclear. We speculate that they might be related to gallstones moving, the damage of pancreatic acinar cells caused by mitochondrial dysfunction, and drug-induced pancreatitis, which requires further investigation.

### 4.2 Hepatobiliary system

Ursodeoxycholic acid is currently the first-line drug for the treatment of cholestatic liver disease and other cholestatic diseases. However, previous studies have shown that patients with advanced liver disease may experience sudden elevation of bilirubin and aggravated cholestasis after UDCA treatment, which can be partially recovered after stopping treatment (33). In addition, studies have shown that patients treated with high doses of UDCA (28–30 mg/kg/day) have twice the risk of cirrhosis, esophagogastric varices, cholangiocarcinoma, liver transplantation, and death compared with patients receiving placebo (34, 35). Consistently, our study also found that these adverse events were associated with the use of UDCA. The reason may be that after exogenous UDCA supplementation, some UDCA that have not absorbed by the small intestine passes through the colon and is converted by the bacteria in the colon into toxic hydrophobic bile acids such as rock cholic acid, which is mostly insoluble in the colon contents and has potential hepatotoxicity and cholestasis promoting effect, and may even react with sulfate to cause liver failure. In addition, UDCA can also induce DNA strand breakage through its unique co-mutagenesis, leading to segmentary bile duct injury, hepatocyte failure and death. Our study also show that long-term use of UDCA was associated with the risk of venous obliterating liver disease, bile duct disappearance syndrome, bile duct stenosis, bile duct obstruction, etc, this has never been reported in previous studies. Therefore, it is suggested that for patients with jaundice with decompensated cirrhosis, UDCA therapy should be suggested from a small dose, and the liver function of patients should be closely monitored to adjust the dose of UDCA during the course of use, especially in the beginning of treatment. Once the progressive increase of serum bilirubin occurs after treatment, UDCA therapy should be stopped in time.

### 4.3 Skin and subcutaneous tissue

Although UDCA has been currently recognized as effective in relieving pruritus in patients with PBC, pruritus was still the AEs associated with skin and subcutaneous tissue with strong signal in our data, and may also be related to the transformation of UDCA into toxic bile acids such as lipocholic acid after entering the human body. Therefore, for patients with obvious pruritus, UDCA treatment should start at a low dose and gradually increase to the optimal dose, especially in the initial stage of treatment (36). Consistent with previous reports, our study also found that erythema multiforme, rubella, lichenoid rash, alopecia, lichenoid drug eruption, and systemic fixed drug eruption were associated with the use of UDCA (37), these results further confirmed the accuracy of our data. In addition, our study also found that some patients treated with UDCA may develop more severe skin reactions, such as pemphigoid, dermatitis exfoliative, toxic epidermal necrolysis, stevens-johnson syndrome and so on. Possible mechanisms involve type 2 immune response, which has been confirmed mainly involved in the regulation of allergic reaction and helminth immunity. Studies have found that Th2 cells and their secreted type 2 cytokines (IL-4, IL-5, IL-10, and IL-13) were the core driving factors of type 2 immune response (38). Exogenous administration of UDCA could regulate the proportion of T lymphocyte subsets, activate Th2 cells and promote the expression of Th2 cytokines (30), thus inducing skin allergic reactions and even severe allergic reactions, this has not been mentioned in previous studies. Therefore, for patients treated with regular UDCA, especially patients with hypersensitivity, we need to be vigilant about the occurrence of skin reactions.

### 4.4 Blood and lymphatic system

Pancytopenia, febrile neutropenia, aplastic anemia, and thrombocytopenia were the hematological AEs associated with UDCA identified in our study, and they have also been reported in a small number of previous studies. The possible mechanisms involve myelosuppression and immune-mediated increased platelet clearance. So the baseline blood cell level of patients should be referred before the use of UDCA in the future clinic, and the changes in blood cell number should be dynamically monitored during the course of medication, especially for children (39, 40). In addition, we found several new and unexpected signals, such as diffuse intravascular coagulation (DIC), coagulation dysfunction, thrombotic microangiopathy, etc. DIC was closely related to the progression of multiple diseases, and its occurrence may be closely related to other side effects found in our data, such as coagulation dysfunction, thrombotic microangiopathy, and thrombocytopenia. Possible mechanisms related to interference with vitamin K1 metabolism, this was first reported in our research. UDCA could promote the secretion of endogenous hydrophobic bile acids into the duct and inhibit their reabsorption, improve the composition of the bile acid pool, thus inhibite reabsorption of vitamin K1 in the intestine competitively. In addition, UDCA could suppress the FXR-SHP signaling pathway in the intestine, reduces the levels of gut derived hormone FGF15/19, thus promotes the expression of liver bile acid transporters and cholecystokinin, accelerates the frequency of bile acid enterohepatic circulation and bile excretion, ultimately reducing the absorption of vitamin K1 in the intestine and increasing its excretion (41, 42). Therefore, in the use of UDCA, coagulation function was also something that we need to pay closely attention to, especially for some elderly patients or patients who use anticoagulants and antiplatelet drugs together, the pros and cons should be weighed. If necessary, we can use vitamin K1 simultaneously to prevent coagulation dysfunction.

### 4.5 Reproductive system and breast disorders

Our results also showed that long-term use of UDCA treatment may have an impact on the patient’s reproductive system and breast disorders. Female patients may have AEs such as lactation disorder, amenorrhoea, menstruation, irregular vaginal hemorrhage, breast engorgement and nodule formation and so on; while male patients may have erectile dysfunction, haemorrhagic cystitis, prostatomegaly, and so on. All these AEs never been reported and should be paid more attention. In addition, the results of previous studies using the FAERS database showed that children aged 0–2 years whose mothers were exposed to UDCA during pregnancy or lactation had stronger AE signals such as syndactyly, infantile or early childhood feeding disorders, and neonatal hypotonia, therefore, it was suggested that pregnant and lactating women should weigh the advantages and disadvantages when using UDCA to minimize the AEs as much as possible (43, 44).

### 4.6 Other related system

There were also some UDCA-related AEs signals in other organs and tissues. Previous reports have shown that UDCA has pulmonary toxicity, which was also reflected in our study (45). For example, the positive signal of interstitial lung disease was relatively strong, and the signal of respiratory muscle weakness, pulmonary interstitial fibrosis were also detected. UDCA also had kidney damage, which could cause tubulointerstitial nephritis, and could also invade the renal parenchyma and cause acute and chronic glomerulonephritis, nephrotic syndrome, renal hypertension, renal fibrosis and other organic diseases of the kidney, these results have not been reported before. Therefore, patients with pre-existing underlying lung and kidney diseases should be vigilant about UDCA-related AEs.

Based on the results of our study, we also found that the frequency of UDCA-related AEs was higher in female patients than in men, which may be attributed to the fact that the main indications for UDCA were more common in women, and female patients were more concerned about physical discomfort and more easily to seek medical treatment. To more objectively explore whether there were gender differences in UDCA-related AEs, we further conducted gender disproportionality analysis. The results showed that pancytopenia, multiple organ dysfunction syndrome, angioedema, ileus, bile duct cancer, and infections and infestations, such as cytomegalovirus infection, human herpesvirus 6 infection, pneumocystis jirovecii pneumonia and so on could only be seen in men. However, thrombocytopenia, agranulocytosis, iron deficiency anemia, lactation disorder, pruritus, calculus urinary, haematuria, disseminated intravascular coagulation, schizophrenia, type I hypersensitivity, pneumonia bacterial, hypoproteinaemia, and so on could only be seen in women. Therefore, in clinical practice, clinicians need to pay more attention to the differences of AEs in patients with different genders to guide more objective drug use.

## 5 Conclusion

This study revealed a broad range of UDCA-related adverse events, including both expected and novel signals across multiple organ systems. These findings emphasize the need for close clinical monitoring, particularly in female patients and those with hepatic or hematologic comorbidities. However, our study also has some limitations. First, the reports recorded in FAERS database were mainly submitted from European and American countries, which were different from Chinese patients by race and region, and could not well reflect the real situation of AE after Chinese patients use UDCA. Secondly, the data in FAERS database were reported spontaneously and submitted from multiple sources, so it was inevitable that there will be random, missing, underreporting, repeated reporting, incomplete reporting, non-standard reporting, mixed indications and AE reports, etc., which may lead to bias in the study results. Finally, the AE signals calculated by disproportionality analysis can only indicate a statistical association between the target drug and the target AE, but not a biological association, which needs to be evaluated and verified by more clinical trials and epidemiological studies.

## Data Availability

The raw data supporting the conclusions of this article will be made available by the authors, without undue reservation.
